# The Next Upgrade for Surgical Procedure-Based Assessments: Clinical Video Footage

**DOI:** 10.7759/cureus.99266

**Published:** 2025-12-15

**Authors:** Priya Patel, Aarjav Naik, Humza T Osmani, Vineet Batta, Jim Gray

**Affiliations:** 1 Medicine, Queen's Hospital, London, GBR; 2 Trauma and Orthopaedics, Luton & Dunstable University Hospital, Luton, GBR; 3 Orthopaedics, Royal National Orthopaedic Hospital, London, GBR

**Keywords:** medical education technology, medicolegal, procedure-based assessment, training, video recording

## Abstract

Introduction

Video recording to teach and assess both technical and non-technical skills is well-established within medical education. Trainees’ clinical and practical competencies are evaluated using procedure-based assessments (PBAs). However, there is limited research describing how these PBAs truly reflect trainee performance.

Objectives

We sought to quantify timing and consultant involvement in PBA completion among orthopaedic trainees, explore trainees’ views on intraoperative video for reflection and PBA completion, and summarise key medico-legal and ethical considerations for routine intraoperative recording in the UK.

Method

We surveyed orthopaedic trainees in the East of England Deanery, United Kingdom. This is a pilot study to highlight the limitations of current systems of PBAs and to explore the potential utility of videos in PBAs. The survey was developed as a pilot by the authors of the study with the consultation of the senior author and pretested amongst the trainees of one hospital before the actual survey was distributed. A six-item questionnaire was then sent out to all trainees of the deanery in online and paper form during face-to-face deanery teaching and to email addresses recorded with the deanery. The responses were recorded and collated directly in the case of paper forms and via a reliable survey platform for the online responses.

Results

The survey response rate was 75% (55/73). Thirty-three trainees (60%) felt that current PBAs do not allow them to highlight their strengths and weaknesses, 48 (87%) felt that retrospective access to a video recording would aid reflective practice, and 39 (71%) felt it would assist with PBA completion. Twenty-seven trainees (49%) reported not completing their PBAs with their consultant.

Conclusion

This paper highlights potential limitations in existing forms of trainee assessment and feedback. We suggest using trainees’ clinical footage to evaluate skills and performance and enhance feedback in PBAs, which has resonated well with trainees, the intended beneficiary. We discuss in detail the medicolegal implications of cameras in operative training, with possible limitations to their adoption in current practice. While video has potential educational value, further research is required to evaluate practical, legal, and educational impacts before widespread implementation.

## Introduction

The use of cameras to observe and teach technical and non-technical skills is established [1-4]. Cameras are already used in theatres with arthroscopic or laparoscopic surgeries being recorded by surgeons and retrieved or broadcast for teaching purposes [4]. Surgeons have consequently provided advice for peers regarding equipment preparation, video recording, and editing/archiving within a reasonable budget [1]. However, their use in open surgery remains limited due to practical constraints.

Procedure-based assessments (PBAs) are also an established form of assessing trainees. A large prospective observational study reviewing various objective forms in assessing trainees within speciality training suggested a need to explore the additional value of video recording [5]. Camera footage cannot replace a trainer's direct supervision in theatre, but it can serve as an adjunct to training. Adherence to patient safety would not permit supplanting real-time supervision by trainers in theatre, as they need to be able to take over if and when required. We hypothesised that if a trainee could safely record their work and upload it to secure NHS software, this could support more accurate recall, assist personal reflection and facilitate discussions with their trainers when filling in PBAs, utilising a more efficient way of teaching and structuring feedback and improving the teaching experience [6]. Such footage is not intended to replace trainer supervision but may serve as an adjunct for structured review. However, there are concerns related to privacy, ethical and future medicolegal implications.

We wanted to review how timely PBAs are being filled in, as well as the perceived viability of supplementing the assessment on the Intercollegiate Surgical Curriculum Portfolio (ISCP) using video footage [7]. We present our findings and, in discussion, clarify medico-legal considerations for the use of cameras in theatre. 

To address gaps identified in previous literature, particularly the lack of detailed examination of how PBAs are completed in practice, we designed a pilot survey study. The objectives were to 1) quantify timing and consultant involvement in PBA completion among orthopaedic trainees, 2) explore trainees’ views on intraoperative video for reflection and PBA completion, and 3) summarise key medico-legal and ethical considerations for routine intraoperative recording in the UK.

Previous iterations of this article have been presented as a meeting abstract in the Annual SICOT Orthopaedic World Congress on the 4th of December 2019 and in the Annual British Indian Orthopaedic Society Conference on the 5th of July 2024 as an abstract.

## Materials and methods

Study design

We conducted a cross-sectional survey of orthopaedic trainees in the East of England Deanery, United Kingdom. The study aimed to evaluate the timeliness and structure of PBAs and to explore trainees’ perceptions regarding the potential use of intraoperative video recordings as an adjunct to assessment and reflection. The survey was developed as a pilot by the authors of the study, with consultation of the senior author, to assess the time between procedure and filling in PBA forms, level of consultant input, time to PBA sign-off and views on current PBA methods, operative video recording, and retrospective access to clinical footage. This was pretested informally amongst the trainees of one hospital before the actual survey was distributed. A six-item questionnaire was then sent out to all trainees of the deanery in online and paper form during face-to-face deanery teaching and to email addresses recorded with the deanery. The deanery itself consists of 39 teaching hospitals. The responses were recorded and collated directly in the case of paper forms and via a reliable survey platform for the online responses. The objective was to assess the time between procedure and filling in PBA forms, level of consultant input, time to PBA sign-off and views on current PBA methods, operative video recording, and retrospective access to clinical footage. 

Study population and sample size

All orthopaedic trainees (ST3-ST8) within the East of England Deanery were invited to participate. Invitations were distributed via deanery email and at regional teaching sessions, with two reminder emails sent two weeks apart. Fifty-five trainees out of the total 73 completed the survey, yielding a response rate of 75%. Participation was voluntary and anonymous. 

Study measures

A six-item questionnaire was designed to capture information on the following areas (Table 1):

**Table 1:** Survey questions

Number	Survey question
1	How soon after a procedure do you tend to fill in a PBA?
2	Do you fill in your PBA with your Consultant?
3	According to your ISCP account, what is the average time between your PBA being filled and it being signed off?
4	Do you think the current structure of PBAs allows you to highlight your strengths and weaknesses?
5	Do you think retrospective access to an operative video recording would be beneficial when you are writing reflections about cases?
6	Do you think retrospective access to an operative video recording would help when filling a PBA with your Consultant?

**Figure 1:**
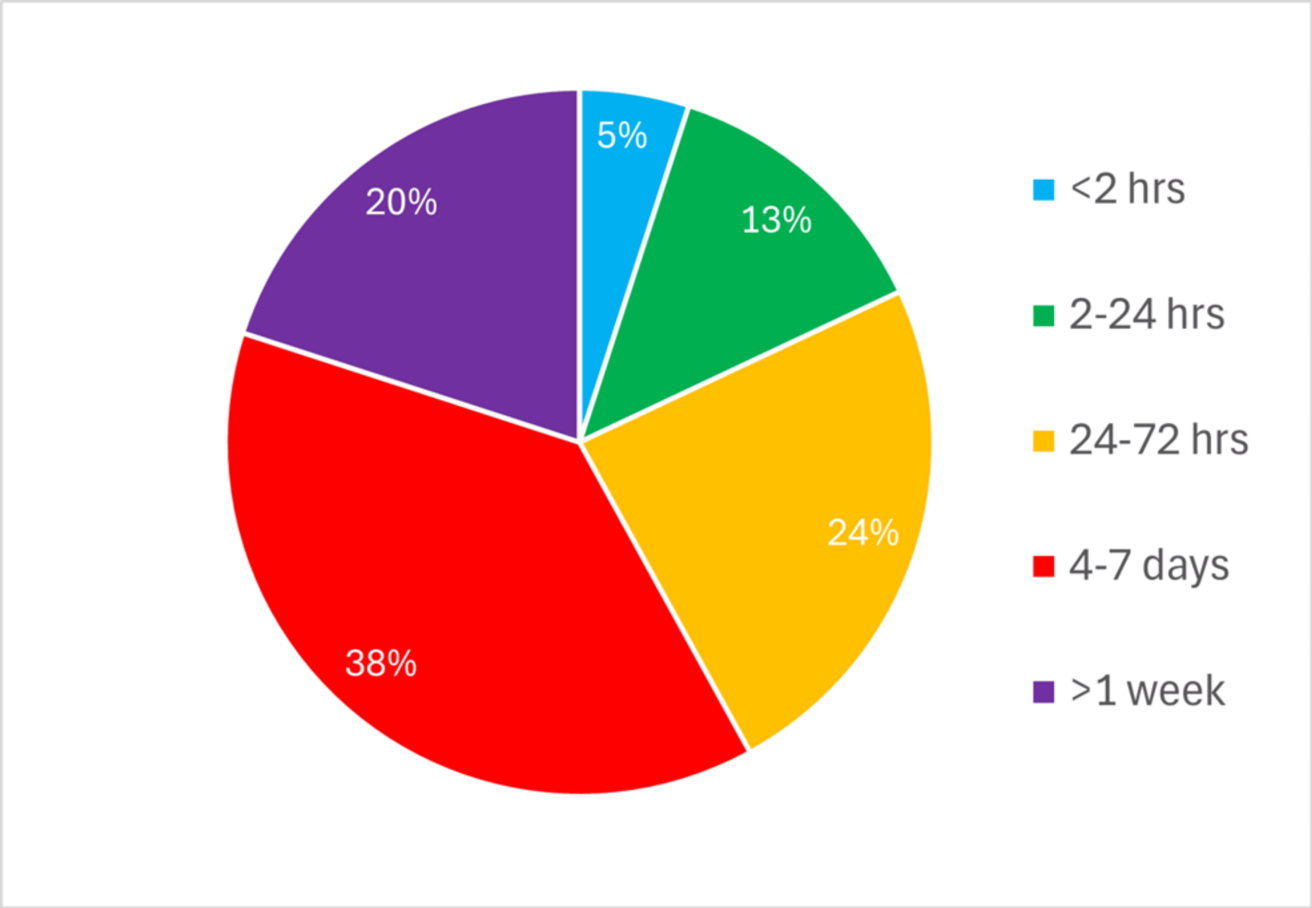
Percentage of trainees responding to Q1 'How soon after a procedure do you tend to fill in a Procedure-Based Assessment?'

**Figure 2:**
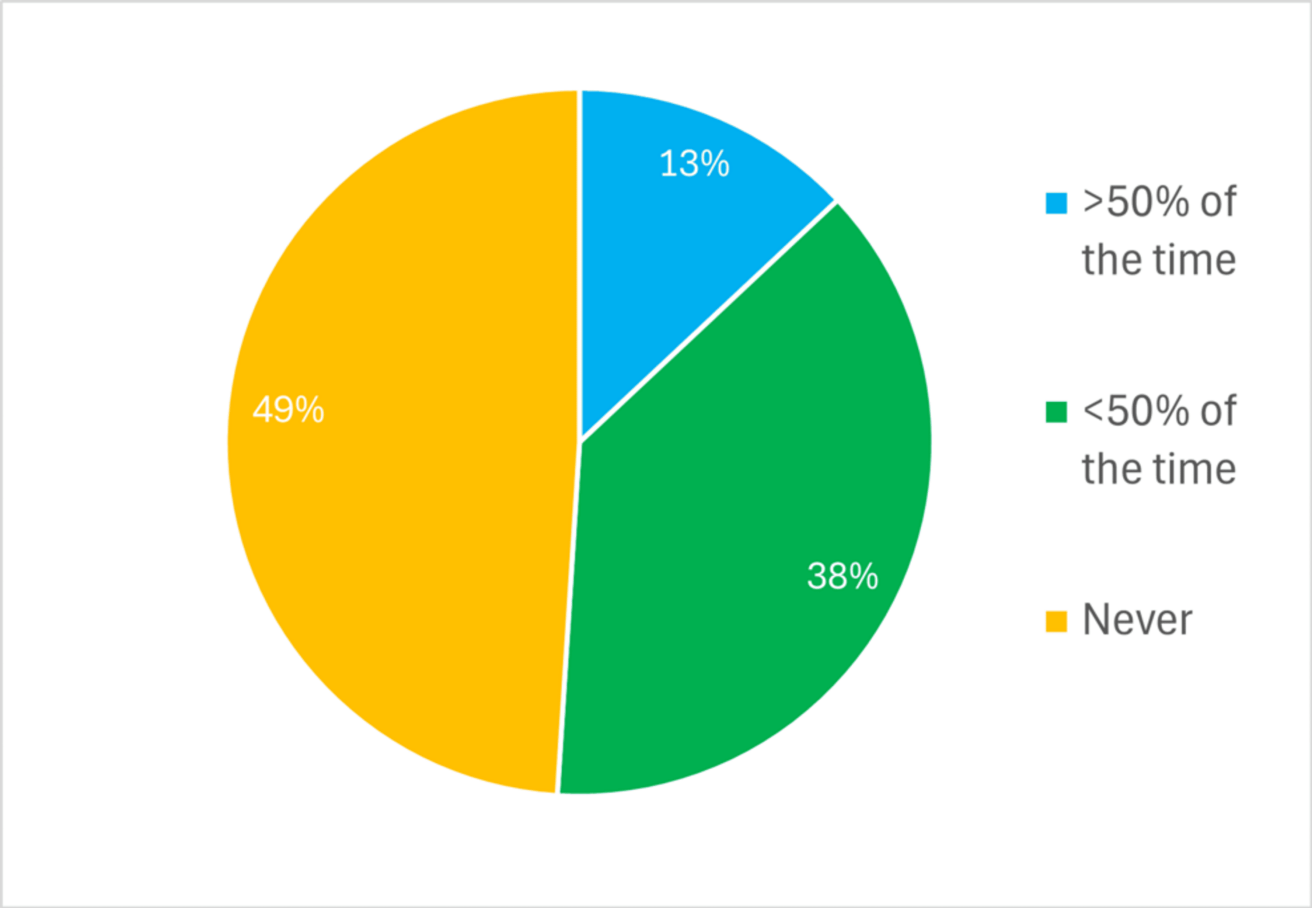
Percentage of trainees responding to Q2: 'Do you fill in your Procedure-Based Assessment with your consultant?'

**Figure 3:**
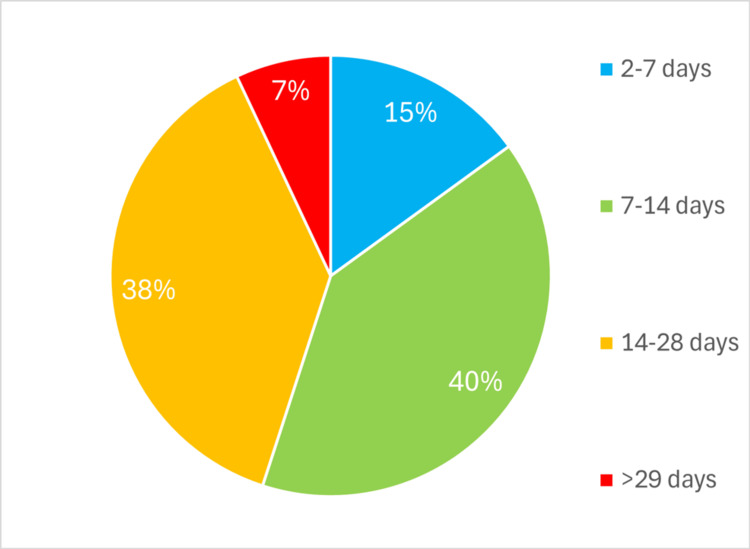
Percentage of trainees responding to Q3 'According to your ISCP account, what is the average time between your Procedure-Based Assessment being filled and it being signed off?'

Questions 1-3 used predefined categories corresponding to the options presented in Figures 1-3 (‘<2 hours’, ‘2-24 hours’, ’24-72 hours’, ‘4-7 days’, ‘>1 week'). Question 2 used a frequency scale (‘never’, ‘<50% of the time’, ‘>50% of the time’, ‘always’).

The participants were also encouraged to provide free-text comments regarding current PBA practices and the use of cameras in theatre for educational purposes for further qualitative impressions.

Training level data (ST3-ST8) was not analysed by subgroup due to small category counts.

Ethics statement

This study did not require formal ethical approval as it constituted an educational service evaluation without patient involvement. All data were collected anonymously, and no identifiable personal information was recorded.

Statistical analysis

Descriptive statistics were used to summarise categorical data, expressed as frequencies and percentages. No inferential statistical analyses were performed, given the exploratory and descriptive nature of the study. This limits the ability to draw causal or comparative conclusions. Results were presented graphically in figures and tables using Microsoft Excel for Microsoft 365 (UK version, Microsoft Corp., Redmond, WA).

## Results

There were 55 respondents to the questionnaire, representing a 75% response rate (55/73). The results for Questions 1-3 (Figures 1-3) were wide-ranging. Only three trainees (5%) completed their PBA immediately (within one to two hours) following a case, 10 (18%) within a day, and 32 (58%) more than three days later. The most common interval was four to seven days, reported by 21 trainees (38%) (Figure 1).

Consultant supervision of the PBA being filled in is outweighed by those compiled independently by the trainees (Figure 2). No trainee reported always compiling a PBA with their consultant. Conversely, 27 trainees (49%) never fill them in with their consultant, and 21 (38%) complete them with their consultant less than 50% of the time. Question 3 (Figure 3) was based on ISCP [6] data; no trainees have their PBAs signed off within a day. Twenty-five (45%) trainees wait more than two weeks, and four trainees (7%) wait for more than a month.

The final three questions were binary ‘Yes or No’ questions (Table 2). Results from these demonstrate that whilst 33 trainees (60%) feel that current PBAs do not allow them to highlight their strengths and weaknesses, 48 (87%) felt that retrospective access to a video recording would help with reflective practice (87%) and 39 (71%) felt it would help in filling in a PBA.

**Table 2:** Summary of results for the binary ‘Yes’ or ‘No’ questions.

	Number (n)	Percentage
Q4 Do you think the current structure of PBAs allows you to highlight your strengths and weaknesses?		
Yes	22	40
No	33	60
Q5 Do you think retrospective access to an operative video recording would be beneficial when you are writing reflections about cases?		
Yes	48	87
No	7	13
Q6 Do you think retrospective access to an operative video recording would help when filling a PBA with your Consultant?		
Yes	39	71
No	16	29

Comments by trainees indicate that their main complaint with PBAs includes the forms being ‘arduous’, ‘long-winded’ and requiring Consultants to write ‘true assessments’. As shown by our data, none of the trainees always fill in PBAs with their Consultants, with the majority often independently compiling them. On the other hand, trainees did feel that any video technology must be easily usable, with the ability to highlight specific parts of an operation. Finally, the most common concern was that of the potential legal implications of any stored footage. These comments represent trainee perceptions only and are not objective measures of PBA quality.

We did not survey the opinions of consultants and trainers because the aim was to see the perspective of trainees and to achieve timely feedback for their portfolios. However, this would be an opportunity for further research and feedback from individuals who have already used recordings for assessment purposes.

## Discussion

Trainee perceptions: summary of findings and implications

The benefits of incorporating video recordings as an adjunct to surgical teaching have been widely discussed. Given the nature of the skills being learnt, certain constraints became obvious from our survey. Delays in PBAs are twofold: the first is due to trainees, with 32 (58%) respondents submitting more than three days after the procedure, and the second is because 25 (45%) reported trainers filled out the feedback more than two weeks following the procedure. The lack of timely feedback can consequently affect the accuracy and specificity of consultant feedback, potentially diminishing both educational value and patient safety-related learning.

The minute details of procedures, which are vital for the reflective writing of the trainee and for feedback by the trainer, can be eroded from memory with time. The memory related to a particular procedure is unlikely to be a memory of a lifetime, as both the trainee and trainer would have experienced it numerous times. Details of everyday experiences, like a routine surgery for an orthopaedic consultant, are not remembered with great accuracy, provided they were not associated with an extraordinary event. Evidence showed that recall of detailed procedural events declines significantly within days to a week. This was seen in a quantitative study on how well human subjects remember the details of specific events occurring during one hour of their lives [8]. The episodic memory focusing on minute details of a procedure is likely to decay over time, for example, the technique of intraoperative placement of implants or the meticulousness of soft tissue dissection or closure. Andermane et al. have proven that recall of detailed memory deteriorates significantly after one week, with accuracy dropping from 74.6% to 58.2% [9]. 

A video of the procedure assists with accurate reflective writing, as well as the trainer providing better-tailored feedback. Reviewing their performance outside of the time-pressured and high-intensity environment of the operating theatre can allow trainees to appreciate details they may not have acknowledged in the moment itself [10]. This, however, does not comment on improving the timeliness of PBA completion but highlights that feedback from video, whenever completed, has the potential to be more tailored and accurate, therefore more likely to address skill gaps. This presents another opportunity for trainees to see their personal progress and learning curve through sequential videos and feedback, providing a longitudinal picture of skill acquisition [11]. Repeated review of footage helps track technical progression, which supports deliberate practice and accelerates skill acquisition in procedural training. However, potential benefits remain theoretical in our pilot study as we captured perceptions rather than performance outcomes.

The Royal College of Surgeons have published 'The Future of Surgery: Technology Enhanced Surgical Training Report', which highlights the potential use of video feedback for safely shortening the learning curve associated with surgery and as an adjunct to training and assessment of performance to progression [12]. The Laparoscopic Surgery Video Educational Guidelines set out agreed components required in using surgical videos for education purposes. They recommend that 'routine video-recording of the procedure and review with feedback sessions should be mandatory in every training program' [13]. Our findings complement these recommendations by demonstrating substantial trainee interest in integrating video into reflective practice and assessment, though formal evaluation of its educational impact is still required.

Another advantage of video recordings is linked to the apparent lack of contact between the trainee and consultant, shown by the survey reporting 49% of the PBAs being filled out without input from the consultant themselves. This is often due to the lack of protected free time for assessments and the time constraints consultants face due to numerous clinical tasks. Video review might provide an additional or alternative opportunity for trainer-trainee interaction, but our data does not confirm whether this would increase engagement or efficiency. A randomised controlled trial showed that feedback following analysing recordings was not quantitatively different from standard feedback and could therefore be used as a reliable and more time-efficient method to provide detailed feedback [14].

Our recommendation on the practicality of creating these recordings is to use a head-mounted camera worn by the operating surgeon. The recording can start prior to scrubbing up and end following closure. For time-efficient review of footage for assessing skills, the trainee or trainer can fast forward to relevant sections of the video without having to watch it all.

A large cross-sectional study about the use of PBAs in the UK highlighted shortcomings of the current system of PBAs, leading to significant deviations from its guidance on use [15]. This included probity issues such as pre-filled comments by trainees instead of the trainer’s comments, sharing of passwords by trainers, etc. This deviation was significant in proportion, as 90% of the respondents reported misuse of PBAs. There was a strong consensus for modification of the current PBA system to make it more ‘real-world’. We believe the use of video feedback can tackle some of the challenges described in this study. It makes the process more transparent and deters irregularities related to probity.

Medicolegal and ethical considerations

The use of footage in any medical field of work begs the following ethical questions: Who owns the video? Who is responsible for any edits or erasure of data? Can patients request a copy to assess a surgeon’s performance? Can the footage be used in any medicolegal proceedings? These medico-legal considerations are interpretive and based on existing national guidance rather than being empirically derived from our survey data.

In available literature on the topic, these issues have not been discussed at length. The RCS FOS:TEST report also raises the issue of patient consent for the same, and rightly so, as there is no national recommendation on the subject [12]. We have done an initial discussion on the subject, considering available guidance from the General Medical Council (GMC) & NHS England. The key aspects of NHS England guidance provided would be clinical appropriateness or indication, digital continuity, storage of data and transparency across the entire process [16].

One concern raised was whether the capture of video and subsequent clipping of footage was acceptable, and if this could be perceived in a litigation case as destruction of potentially viable prosecuting evidence. In accordance with guidance and in the interest of transparency, recordings must not be edited and should be kept in their original form. One could add edits to the education portfolio element, so long as there remains a permanent store of all the captured footage, which will need to be accessible in the electronic patient record (EPR). Therefore, it would be prudent that the surgeon has total control over what they wish to capture, which would require switching (record/pause by hand/foot/voice recognition), which we would suggest would necessitate a slick user experience.

The consideration of this from a software perspective is that all footage is passed to the medical record with edits retained within a software portfolio. The EPR software already supports video upload and is either already being used or is under consideration in most NHS trusts. Other options for storage are cloud services being used by IT services within the trust. This would also be keeping in line with the NHS England and GMC guidance emphasis on digital continuity, security, and anonymity.

We believe it would be unwise to edit the video before uploading. If any editing or curating is done before upload, this would be at the discretion of the editor, who is likely to be the trainee. Expecting the consultant to do so before uploading and reviewing with the trainee defeats the purpose of finding a way to minimise the effect of workload and time constraints. It then becomes counterintuitive to edit the video without both parties involved, as what might be important for one might not be for the other, particularly when the purpose is for feedback and assessment. For example, when performing a hip replacement, if we edit out the positioning and include only operative portions of the footage, the trainer might not be able to give feedback about positioning, which is still important for trainees to consider. This practice of uploading the whole video would ensure that no minute details are deleted and able to be reviewed as appropriate for both trainee and trainer.

The possibility of use of the footage in a medico-legal proceeding can make surgeons sceptical regarding whether the utility outweighs the increased litigation risk due to increased fear of a ‘big brother environment’. However, the risk is not new to orthopaedic surgeons as there is a similar risk with intraoperative fluoroscopy images, postoperative radiographs and intraoperative clinical radiographs, etc. A centre that used video recordings within the emergency medicine department for 11 years noted no medicolegal subpoenas or issues relating to employment and liability during this period [17]. Conversely, the said footage can also be used to demonstrate that all possible efforts were made intraoperatively to tackle any complications, and there was no professional negligence.

The footage obtained as 'part of the patient’s care is part of the medical record and must be treated in the same way as other medical records' [18]. This can be stored on secure NHS software like other evidence, such as radiographs and electronic patient records. The trainee and the assessor must access the footage from secure NHS software only for the purpose of feedback. We think this would address any confidentiality concerns. It must be retained for eight years according to the advice from the NHS England on records retention, which should be followed [16].

Another consideration while selecting the video format and the recording device is digital continuity. The selected format must last at least for the retention period and preferably more. If one is planning to implement this on a wider scale, attention should be paid to uniformity across different sites. This would avoid learning a new system whenever one moves to a different trust. This could be a recurring problem leading to low compliance as trainees rotate through different NHS trusts on a regular basis. Lastly, the clinical appropriateness of the footage needs to be decided by the organisation. It should be determined whether the recording is only for teaching purposes or is part of the provision of healthcare.

Consent must be obtained and clearly indicate that the purpose is for the medical record and medical education. This can be achieved through amending the consent form to include the same or adding a separate consent. Doctors need to consider that 'when used for clinical purposes, such recordings form part of the patient’s medical record and the same standards of confidentiality and requirements for consent for disclosure apply' [18].

For recordings made for secondary purposes such as teaching, research, or assessment, the GMC requires clear agreement on ownership and intellectual property. Doctors must explain why a recording is being made, obtain valid and pressure-free consent, and stop recording if the patient asks. Identifiable recordings can only be used with consent or legal authority, must be stored securely, and handled according to relevant laws and local policy. Anonymised images may be used for education or research without consent, but clinicians should still inform patients where possible and document the discussion. Any recordings that may contribute to patient care and are not anonymised generally require explicit consent. [18].

Another issue is whether the ability to record then mandated a recording, or whether recording remained a choice. The entirety of what is captured must be subsequently stored and not permanently edited out. It is acceptable to have video equipment available, but not used.

Beard et al. identified a mixed response to the use of PBAs for assessing trainees in theatre; our study supports their findings with a range of responses regarding how beneficial trainees feel these forms are in representing their progress [5]. Furthermore, they recommended the need for an assessment of cameras in training.

It is worth noting that the idea of using an 'OR Black Box', which records audio and panoramic video inside an operating room, has been getting traction lately in Europe and North America. The idea is to comprehensively capture data to find out hazards before accidents occur [19]. This can identify environmental distractions, variation in the surgeon's skills and other factors associated with an adverse event in theatres. This improves on current methods like retrospective analysis of self-reported data from morbidity and mortality rounds, incident reports, and patient charts when evaluating adverse outcomes. Given that this method of analysis has already been implemented for patient safety and monitoring adverse events, there is an argument that this can then be used for medical education purposes.

Artificial intelligence (AI) is becoming increasingly used throughout education and assessment. We can integrate this within future training methods in order to give more objective and structured feedback using AI learning systems. A recent study compared an AI tool trained on laparoscopic colectomy videos against expert human raters who traditionally grade PBAs or video-based assessments. The ratings correlated strongly with the expert evaluations, highlighting the potential feasibility and validity of using AI as an adjunct in surgical video-based assessment [20]. A systematic review of using AI for assessment of technical skills in minimally invasive surgeries showed good performance by AI, but there is still a need for further research into how the algorithms should be modelled to ensure validity and generalisability outside of specialist centres [21]. A pilot study has investigated the role of AI in feedback and concluded that automated video analysis helped improve the performances of novices practising robot suturing underachievers [22]. However, there is much research to be done before this can potentially be extended into an analysis of major operations and then also be used to aid structured feedback for developing trainees. This was outside the scope of our pilot study and what our survey evaluated.

Limitations

This study has some limitations. First, although the response rate was relatively high (75%), the study represents a convenience census sample of trainees within a single UK deanery. This limits the generalisability of findings to other regions, specialties, or training structures, as well as highlighting a potential selection bias. Second, the study relied on self-reported survey responses, which are subject to recall and response bias, particularly regarding the timing of PBA completion and consultant involvement. These results describe trainee experiences and opinions, not objective measures of PBA quality, feedback accuracy, or the effectiveness of video-assisted review. Third, the survey did not capture consultant perspectives, which could provide a more balanced understanding of current assessment practices. We have also not commented on how trainee and trainer familiarity with technology could influence the response. Finally, as a descriptive analysis, the study did not include inferential statistics, so the findings should be interpreted as exploratory rather than definitive.

Despite these limitations, this study addresses an important and underexplored aspect of surgical training, which is whether current workplace-based assessments accurately capture trainee performance and how technology could improve this. The high response rate and consistent trainee appetite for video-assisted reflection demonstrate that the issue is both relevant to current training practice and of practical importance for future curriculum development. Notably, since the study, cameras have been installed in the light handles of canopy lights of theatres for this purpose within one of the hospitals within the deanery (Luton and Dunstable Hospital). This highlights the need for further research in this area, including incorporating consultant perspectives, multi-centre sampling, and mixed-methods evaluation to help establish the broader applicability and educational impact of video-assisted PBAs.

## Conclusions

Our pilot study has suggested an appetite for the use of cameras in theatre to improve the ability of a trainee to reflect and discuss a case with a trainer. Our findings reinforce existing concerns regarding the accuracy and educational value of delayed workplace-based assessments. However, legal and ethical implications, including the need to store all the data and ensure compliance with existing standards, the potential of being used in a medicolegal proceeding and the cost implications, do potentially affect the ease with which this may be implementable. These concerns could preclude the rapid adoption of the use of footage for teaching. Trainees demonstrated clear interest in using intraoperative video to support reflective practice and facilitate more specific feedback, but while video recordings may offer educational advantages, our findings do not provide objective evidence of improved performance, timeliness, or assessment quality. While we believe there is potential benefit, future work is required to evaluate the practical, legal, and educational impact of such systems in real-world settings. The benefit seems more than the risk, corroborated by the fact that video has been increasingly used in the interest of patient safety and teaching. Whether it is adopted in UK practice as an adjunct to current assessment and feedback systems remains to be seen.

## Figures and Tables

**Figure 4:**
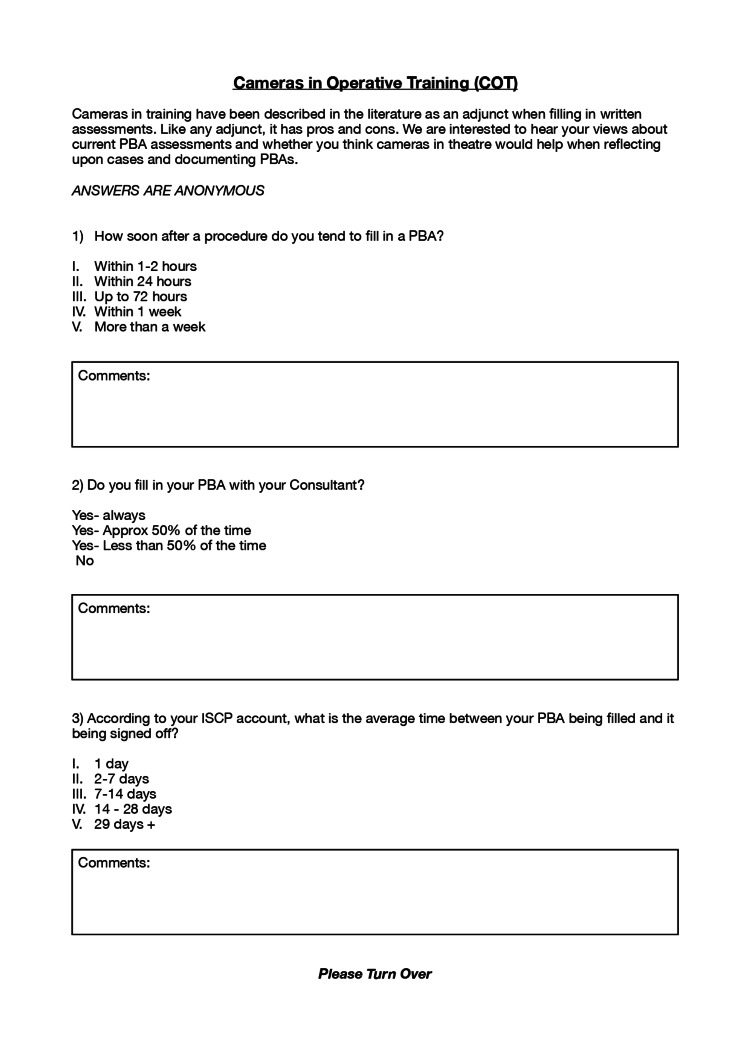
Cameras in Operative Training (COT) questionnaire page 1

**Figure 5:**
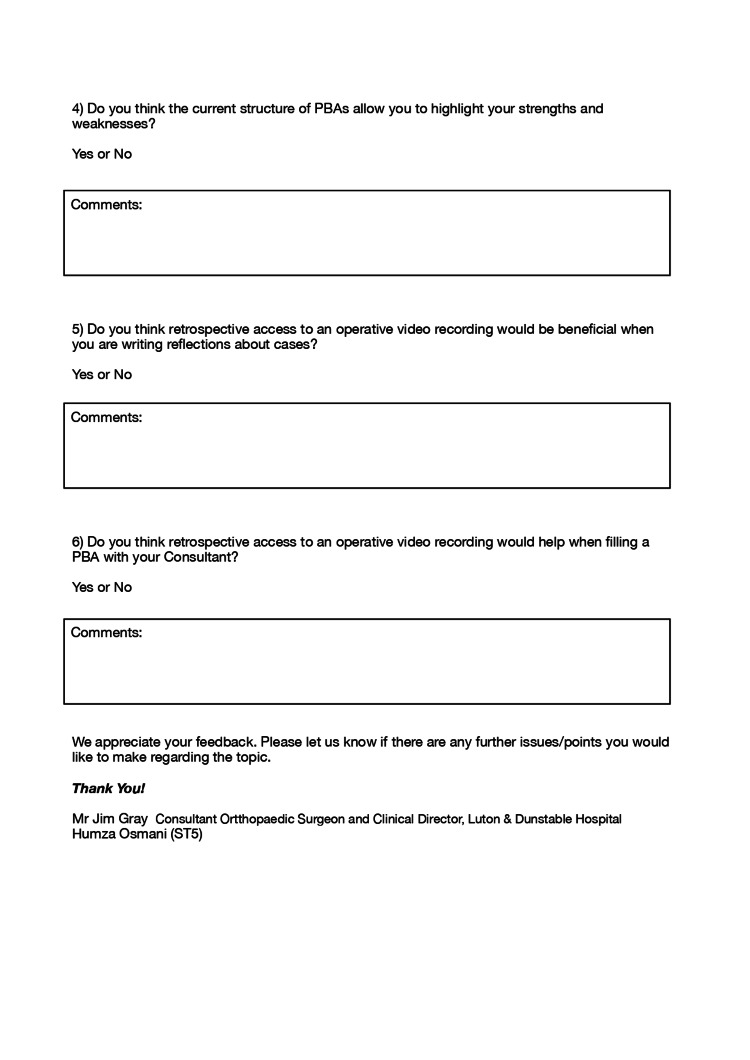
Cameras in Operative Training (COT) questionnaire page 2
